# Assessment of Augmented Reality in Manual Wiring Production Process with Use of Mobile AR Glasses

**DOI:** 10.3390/s20174755

**Published:** 2020-08-22

**Authors:** Andrzej Szajna, Roman Stryjski, Waldemar Woźniak, Norbert Chamier-Gliszczyński, Mariusz Kostrzewski

**Affiliations:** 1 DTP Ltd., 66-002 Zielona Góra, Poland; a.szajna@dtpoland.com; 2 Faculty of Mechanical Engineering, University of Zielona Góra, 65-001 Zielona Góra, Poland; r.stryjski@iizp.uz.zgora.pl (R.S.); w.wozniak@iizp.uz.zgora.pl (W.W.); 3 Faculty of Mechanical Engineering, Koszalin University of Technology, 75-453 Koszalin, Poland; 4 Faculty of Transport, Warsaw University of Technology, 00-662 Warsaw, Poland; mariusz.kostrzewski@pw.edu.pl

**Keywords:** augmented reality, industry 4.0, wiring, assembly, production, AR glasses, AR headset, human-machine interaction interface, switchgear, control cabinet

## Abstract

Digitalization of production environment, also called Industry 4.0 (the term invented by Wahlster Wolfgang in Germany) is now one of the hottest topics in the computer science departments at universities and companies. One of the most significant topics in this area is augmented reality (AR). The interest in AR has grown especially after the introduction of the Microsoft HoloLens in 2016, which made this technology available for researchers and developers all around the world. It is divided into numerous subtopics and technologies. These wireless, see-through glasses give a very natural human-machine interface, with the possibility to present certain necessary information right in front of the user’s eyes as 3D virtual objects, in parallel with the observation of the real world, and the possibility to communicate with the system by simple gestures and speech. Scientists noted that in-depth studies connected to the effects of AR applications are presently sparse. In the first part of this paper, the authors recall the research from 2019 about the new method of manual wiring support with the AR glasses. In the second part, the study (tests) for this method carried out by the research team is described. The method was applied in the actual production environment with consideration of the actual production process, which is manual wiring of the industrial enclosures (control cabinets). Finally, authors deliberate on conclusions, technology’s imperfections, limitations, and future possible development of the presented solution.

## 1. Introduction

The current world-class challenge is to sustain or increase production capacity of a higher quality with fewer inputs in order to reduce time, cost, and equally enhance planning productivity (Navaei and ElMaraghy, 2018 [1]). Modern production, with constant pace for overall improvement through an increase of process automation and digitalization, prompts increasing demands on high-tech products and services for manufacturing. As a response, new highly specified methods and techniques keep on arising to support management, production, and logistics processes, and importantly, the work of shop-floor employees (Syberfeldt et al., 2016 [2]).

In every industry, regardless of the implementation of the Industry 4.0 ideas (Gilchrist, 2016 [3]), which lead to significant automation of production or logistics processes and operations, manual activities, depending on people’s operations, are always of high importance. Therefore, apart from robotization and automation, researchers’ thoughts toward improvement should always take human demands into consideration.

As noted by scientists from the German Research Centre for Artificial Intelligence (DFKI—Deutsches Forschungszentrum für Künstliche Intelligenz), a significant research area in the Industry 4.0 topic is to find the most convenient human-machine interaction interface (Gorecky et al., 2014 [4]). Taking the abovementioned fact into consideration, it is worthwhile to quickly introduce the importance of human-machine interaction interfaces. This introduction relates to different research areas as well as to various industrial sectors. The indirect relation is connected, for example, to the automotive industry, and directly related is the connection to robotics as a special interest of Industry 4.0. Nowadays, interdisciplinary studies on the abovementioned topic may intersect in different research fields; therefore, selected publications presented below are an introduction to the main subject matter of the article. The first example is the human-computer interaction system connected to eye movements and finger tracking technologies in the automotive industry as described in Zeng et al. (2019) [5] (eye movement tracking is understood here as directly corresponding to technologies presented in this paper). Moreover, Zhao et al. (2018) [6] researched and described the human-machine interaction interface for the dispatchment automation system. Visual analytics mentioned in the quoted paper are treated by this paper’s authors as an important aspect for the development of any kind of system where the correspondence between human and on-line tools exists. Visual perception is also important in robotics, where the human-machine interaction interface was researched in connection to the sensing intensity of cone cells by Zhang et al. (2016) [7]. This matter is highly connected to cognitive ergonomics, which is mentioned in Deng et al. (2016) [8] who took into consideration the design of the driller control room on a drilling rig. In order to analyze a human-machine interaction interface, authors of [8] used a genetic algorithm and ant colony algorithm in their research. Eye movement tracking, visual aspects, and cognitive ergonomics, to name a few, have significant importance to a system considered in this paper.

One of the tools which have been gradually introduced in order to influence the efficiency and quality of the human-machine interaction interface is the augmented reality (AR) headset, or simply, the AR glasses (Azuma, 1997 [9]; Pereira et al., 2019 [10]). Pereira et al. (2019) [10] mentioned that AR glasses (smart glasses) are one of the most common applications of AR technology, next to smart helmets when wearable devices are based on head-mounted displays (HMD) are taken into consideration in the industrial environment. As Ro et al. (2018) [11] defined, the AR glasses are “devices that are worn like regular glasses and merge virtual information with physical information in a user’s view field”. This kind of headset featuring efficient head-anchored tracking solutions was feasible by an increasing capability of computing power and mobile graphics (Cutolo et al., 2020 [12]).

The AR applications are presently used in numerous domains, ranging from industrial engineering to clinical services, field repairs, and lifestyle management (Choi et al., 2019 [13]). From the perspective of this paper, industrial engineering applications of AR headsets are of the highest importance. However, it is worth mentioning that the AR headset can be utilized in pharmacology and medicine as well. The employment of this kind of equipment in the abovementioned fields was researched by Li et al. (2020) [14] in regard to supporting people with hearing disabilities. Moreover, considering the same research field, Ingeson et al. (2019) [15] researched medication coach intelligent agents aided with the AR headset that could manage different types of information such as medication regime and restrictions of medical patients as well as patients’ preferences. Patients in the ophthalmology clinic were investigated with the use of AR headset solutions as well by Ong et al. (2019) [16]. Also, molecular analyses were conducted with the use of the AR device, for example in the study of Müller et al. (2018) [17]. Selected solutions were investigated in agriculture. For instance, AR visualization was applied to agricultural fieldwork navigation in Zheng and Campbell (2019) [18].

Recently, the AR glasses with a noninvasive single-channel brain-computer interface has been analyzed as a wearable monitoring system for inspection in the framework of Industry 4.0 (Angrisani et al., 2020 [19]; mentioned in Govindarajan et al., 2018 [20] as well). The AR glasses might be also used as an aid in the initial training of students and young employees. For industrial engineering purposes, the AR headset was researched to enhance the ability of superintendents to perform infrastructure inspections, mapping infrastructure on the site, generating models of inspections, and providing compliance between various superintendents as in Ballor et al. (2019) [21]. Authors of this research stated that despite structural health, monitoring is recognized as the key discipline to evaluate the integrity of structures as non-hazardous research, human-based visual inspections remain the dominant method of work, making the AR a valuable technology for that purpose. With a reference to the robotics in the context of Industry 4.0 as mentioned in the previous paragraph, classic bespoke research was presented in Angrisani et al. (2018) [22]. The AR headset in this research was used in order to enhance and assess human-robot interaction, driven by steady-state visually evoked potentials (SSVEP) non-invasive single-channel brain-computer interface. Additionally, in the subject of robotics, the research connected to the application of AR aiding an operator control panel of a teleoperated mobile robot was investigated in Kot et al. (2018a, 2018b) [23,24].

The other common area of interest is the application of AR in shop-floor management. Shop-floor tasks such as maintenance, repair, or assembly are still identified as strategic AR application fields (Lamberti et al., 2014) [25]. In the research by Ens et al. (2016) [26] (cited after Lee and Hui, 2018 [27]), a wearable human-machine interaction interface is mentioned, however, the challenge concerning this interface is connected to the fact that it must be ready for mobility or in situ use. At the same time, the literature review showed that the human-machine interaction interface is not a common solution in design, assembly, and use of an electrical control panel, known widely also as a control cabinet or switchgear system. In order to develop such a solution, one might use AR glasses, as applied in this paper.

The importance of the potential in using AR glasses has been identified by several research institutions. For example, researchers from the University of Oxford and Graz University of Technology noted that the effects of AR applications on key performance process indicators are sparse (Vorraber et al., 2019 [28]). The usability evaluation and testing of such applications is a persistent topic discussed on a regular basis during various symposiums and conferences on AR, including one of the most significant ones, such as the International Symposium on Mixed and Augmented Reality conference, where usability evaluation and testing was pointed in the first decade of 21st-century trends’ review in Zhou et al. (2008) [29] and prolonged for the next decade in the review by Kim et al. (2018) [30].

The abovementioned, brief literature review involves various solutions and research on AR technology. These topics have been indicated, in order to emphasize the validity of the application of mentioned solutions and, at the same time, the diversity of their applicability. The review of the literature indicates a deficiency in research and publications related to some applications of AR technology. The application of AR is one of the core interests of the authors, especially in regard to control cabinet assembly.

One of the most significant topics in AR is creating real-life applications. High interest in various areas connected to production can be observed. In the case of this research, the process of wire, wire harness, and cable assembly are considered. The research in this field has been developed since the 1990s (Hassan and Rahini, 2016, p. 134) [31]. It is noteworthy to mention that the term ‘augmented reality’ was used for the first time in this period by researchers working for the Boeing company on the system supporting the identification of the beams and individual electric wires in the aircraft they were constructing (Lee, 2012) [32]. The AR headsets assured new possibilities in wire assemblage.

The authors of the article noticed the possibility of using AR technology in the distinctive production of control cabinets. The wiring assembly is one of the key elements and the most time-consuming operation of the analyzed production process, which is carried out manually by skilled technicians. The assembly is based on repeated manual activities, which are a time-consuming factor in the entire production process. Taking into account the nature of the analyzed process, the purpose of the research presented in the article is to discuss the possibility of using the proprietary support system, based on the AR glasses at the stage of wiring assembly in control cabinets. On the other hand, the research problem is to verify whether the use of the AR system in this process contributes to the reduction of the wiring time and whether in the work of the operator, wearing the AR glasses is not inconvenient, so it would not jeopardize the possible reduction of assembly duration. The authors formulated the following hypothesis: The convenient human-machine interface based on the AR support system should allow a user to save time on performing the manual wiring production process, without causing considerable discomfort or difficulties in performing the task, in comparison to performing the same tasks in the process without using such support system. Application of the AR support system as a solution requires testing and verification, which are essential before driving such a solution to the market.

The first AR demo application, supporting the manual wiring task in the control cabinet production process, with use of newly released AR glasses, Microsoft HoloLens ver.1, was presented as a top innovation by Szajna A. from the Digital Technology Poland company during the world’s biggest industry trade fair as Hannover Messe in 2017. The presentation included a demonstration of the device to domestic authorities of Germany (Chancellor) and Poland (Prime Minister) who were present at the official opening ceremony (DTPoland, 2017) [33].

The solution was described to some extent in 2018, together with a brief description of AR technology (Szajna et al., 2019) [34]. This paper is a continuation of the abovementioned research, enriched with the validation and test study. This contribution presents the results of the comparison of the solution with and without the use of the AR glasses applied in field research.

In Section 2 of this paper, the authors present the run of the wiring process in control cabinet production. In this section, the principles of the AR support system for manual wiring are presented as well. Section 3, Section 4, Section 5 and Section 6 describe the test setup and procedure of the production process study with detailed results of the tests. Subsequently, the technology imperfections, limitations, and future development descriptions are given in Section 7 followed by conclusions in the last section.

## 2. The Evolution of Manual Wiring Production Process from Use of Paper Documentation, Through Touch Screen Application, to the AR Support System

**Figure 1:**
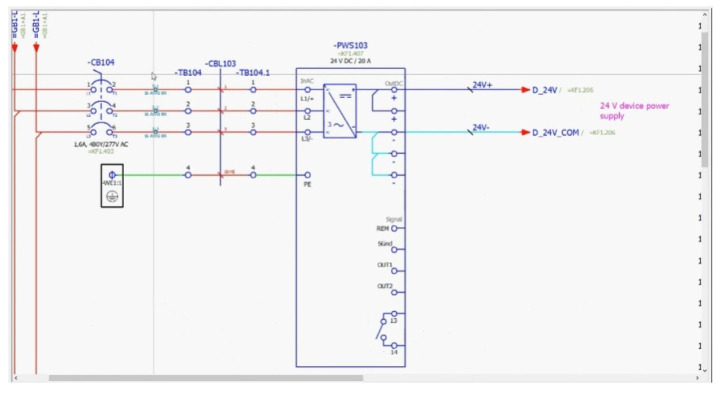
The control cabinet design in a CAD program—EPLAN Pro Panel 2.7 used in this case.

The process of industrial enclosure assembly, which results as a control cabinet, consists of two main steps: (1) the design of an electrical and wiring diagram to fulfill the specification and (2) the assembly of all the parts together. Assembly divides into another two steps, which are (2a) attachment of components and (2b) wiring them according to the designed diagram (wiring scheme). The design of the diagram in the first step is carried out in an E-CAD/CAD system (E-CAD—electronic computer-aided design), also referred to by professionals as EDA (electronic design automation; Bryant et al., 2001 [35]), as presented in Figure 1. E-CAD is known more as CAD (computer-aided design) and as such is recalled further in this paper. It is a category of engineering software tools for the design of electronic systems among which circuits and printed circuit boards are represented. The CAD data, with the example given in Figure 1, are the basis for the AR support system (Hamrol et al., 2019 [36]).

Up to the year 2016, the process of manual wiring and wiring harness assembly was performed based on an extensive paper printed documentation, depicting electric schemes and a way of wire connections (DTPoland, 2017: press release [33]). A highly skilled electrician interpreted page by page and mounted wire after wire. It was observed by Szajna A. during research stays at 20 different control cabinet production facilities, including minor ones, medium size, and big enterprises. Among them all, employees of 18 facilities used printed *.pdf files and another two used also *.pdf files, yet displayed on monitors. All of the 20 facilities used CAD systems for designing, but none equipped the shop-floor employees with any convenient assembly support system. Such a scenario was reported to be observed in many other places by Schall J.H. (2015, personal communication), who analyzed and worked around the topic of control cabinet assembly for several years (Schall, 2010) [37].

**Figure 2:**
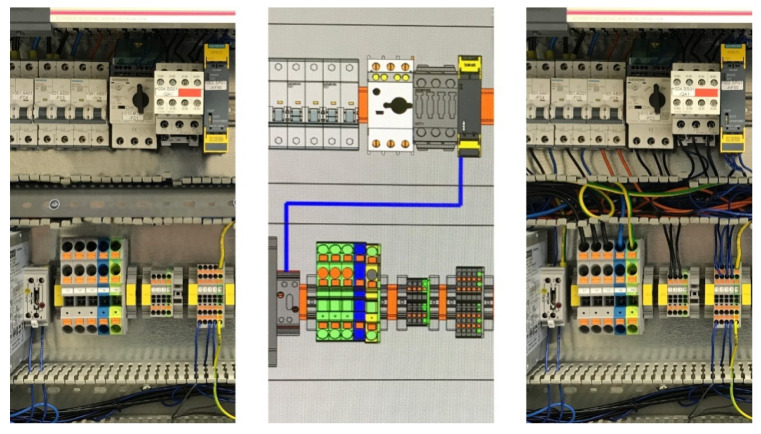
Wire assembly process—left photograph: an industrial enclosure with assembled components; middle graphic: wire assembly instruction for single blue wire (digital twin with the 3D graphics from ESW system); right photograph: wiring completed. Photographs: Szajna A.

Manual wiring has undergone a breakthrough, when Schall J.H. presented the idea of a wiring support system in 2015 during the Hannover Messe trade fair (Phoenix, 2015 [38]; EPLAN, 2015a [39]: starting in 10:45 [mm:ss] of cited video coverage). This revolutionary change connects with the construction of a virtual prototype of an enclosure, the so-called “digital twin” of a real enclosure device (Tao et al., 2018 [40]). The example of such a solution is given in Figure 2.

This figure illustrates the wiring production process, showing a before-wiring picture of an industrial enclosure (left side of Figure 2), and digital twin mirroring to help the user easily understand the design (center of Figure 2) and after-wiring picture enclosure with assembled wires (right side of Figure 2). Later in the year 2015, Szajna A. mentored by Schall J.H., took one step forward and researched, defined, and implemented together with the engineering team the first interactive, touch screen demo for the end-user. It was presented at the SPS Nürnberg 2015 trade fair (Nürnberg, Germany), under the name EPLAN Smart Wiring (ESW) system (EPLAN, 2015b [41]; Rittal, 2015 [42]: starting in 02:50 [mm:ss] of cited video coverage), with the aim to make tests and collect feedback for such a solution.

The transition of the wiring production process from using paper documentation to implementation of the digital support system was a significant contribution to the field of Industry 4.0. The users of this system were provided with the complete digital twin concept, including the 3D graphics for effortless visualization and all the necessary information with a step by step instruction for the whole wiring process. Substantial time savings were reported when using the ESW, which turned out to be a state-of-the-art market solution one year later (EPLAN, 2017 [43]).

**Figure 3:**
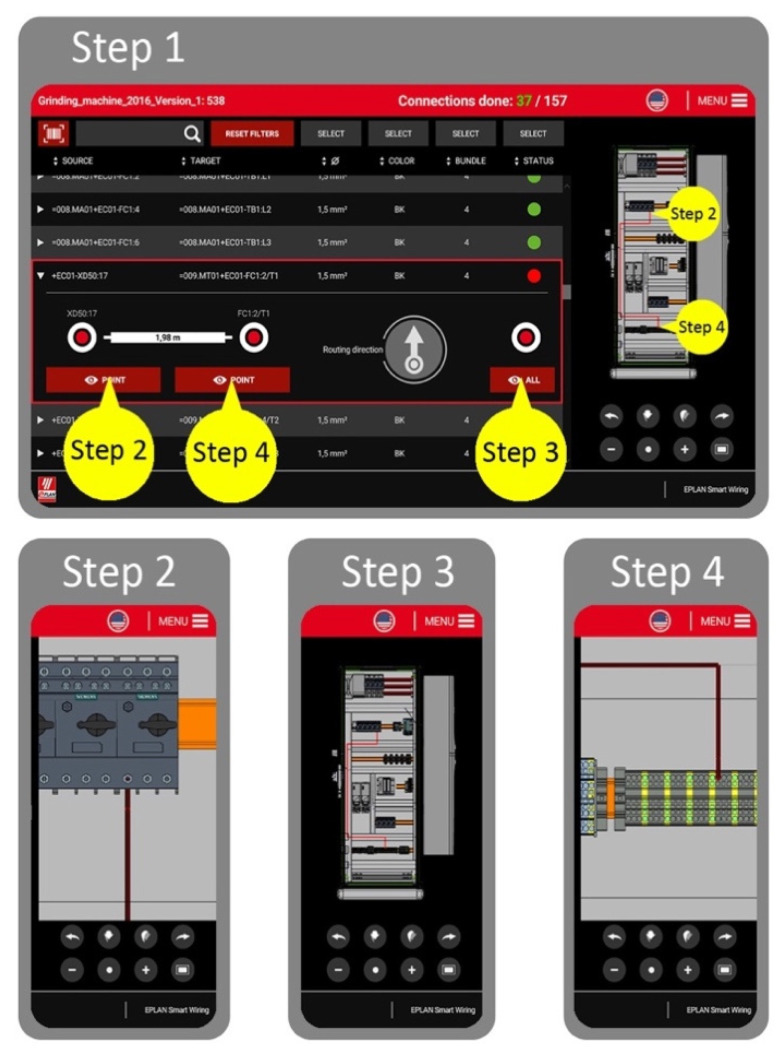
EPLAN Smart Wiring (ESW) application (Step 1) with 3 zooms of the 3D graphics, showing the zoomed connection point (Step 2, Step 4) or whole wiring routing (Step 1, Step 3).

The exemplary 4-step usage of the ESW support system is shown in Figure 3. This operation was included in the research as part of the test procedure (the test procedure is described in Section 4.4., however, it was important to introduce the ESW support system in this part of the paper in view of the further argumentation). Application of the ESW system consists of the following steps:

- Step 1: Selection of a wire to be assembled from the list; reading information and interpreting the 3D graphics of the entire wiring diagram in order to know how to route and assemble the wire.
- Step 2: Zooming in (visualization enlargement) the graphics in order to learn the exact (first) connection point; connection of the first wire end.
- Step 3: Zooming out (visualization reduction) the graphics to recall the wiring route; leading the wire through the wire ducts.
- Step 4: Zooming in (visualization enlargement) the second connection point and connecting the second wire ending.

These four simple steps assure the clarity and ease of use of the application, even for less qualified employees.

Introduction of the Microsoft HoloLens in 2016 (Kim et al., 2018 [30]) provided a tool for further research on specific applications (mentioned in the literature review in this paper).

When the AR glasses became available for developers at the turn of the years 2016 and 2017, Szajna, A. envisioned and designed the method of an AR support system for manual assembly of wires and wire harnesses, based on the previously developed ESW system. Shortly afterward, together with the engineering R&D team, the elaboration of the method’s implementation was launched. It took several more months to develop the prototype, namely DTPoland AR Smart Wiring 4.0 system, with two patent applications EP000003392987 (Schall and Weichsel 2018 [44]) and PL000000421368 (Adaszynski et al., 2018 [45]) for the AR wiring process and wire label reader (DTPoland, 2017 [33]). The latter has recently obtained the status of a European patent EP000003460719 (Adaszynski et al., 2019 [46]), while Schall and Weichsel (2018) [47] recently obtained the US patent for their method of the automated support in a connection process, in particular of a wiring process, of components arranged in a switchgear cabinet (applied for the patent in 2018). As mentioned in the introduction, the first demo of the system was presented during the Hannover Messe trade fair in 2017. 

The DTPoland AR Smart Wiring 4.0 system, presently still in the prototype phase, as aforementioned in the paper by Szajna et al. (2019) [34] (this system also relates to the ESW support system). An important function of this solution is the implementation of interchangeable instructions directly in front of users’ eyes, delivered in online mode, whereby operators’ hands are free from using any stationary device or a paper manual so that a particular operator can conveniently assemble the wires in a control cabinet. The system is discussed in greater detail further in the paper in order to provide a better understanding of the authors’ study.

## 3. The Assumptions, Principles, and Aims of the AR Support System for Manual Wiring Production Process

The system mentioned at the end of the previous section, namely the DTPoland AR Smart Wiring 4.0, is the prototype using AR glasses that enhance the production station with a set of virtual pointers and images (plus a virtual screen, if a regular touch screen is omitted) augmenting the real-world environment. 

**Figure 4:**
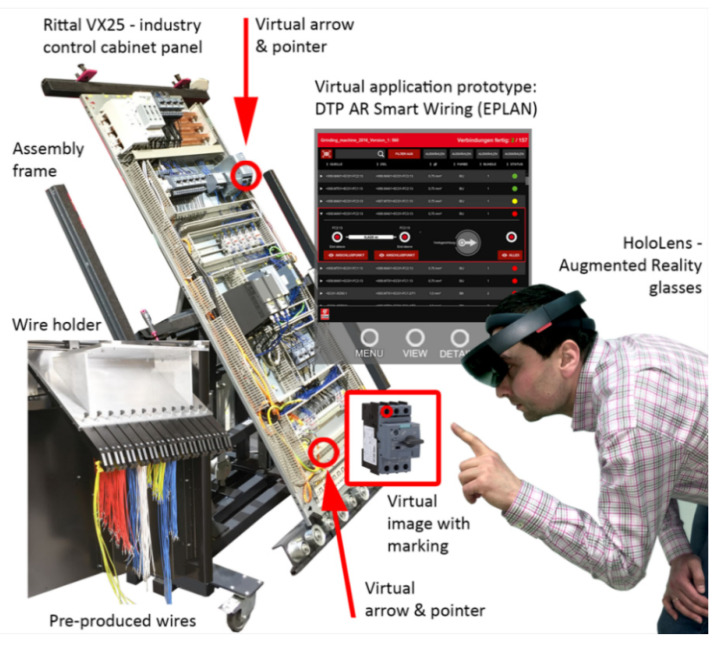
Visualization of the production station with the DTPoland AR Smart Wiring 4.0 prototype system. Source: based on Szajna et al. (2019) [34] with the necessary upgrade included.

The control cabinet production station consists of several devices and the prototype (Figure 4), such as:

- Touch screen with the manual wiring support system (it is a market available solution without the use of any AR prototype).
- Assembly frame.
- Wire holder (Figure 4 presents a prototype; however, an assembly station is always equipped with a holder, therefore it has no influence on the described study).
- Production tools (screwdrivers, etc.).
- The prototype.

It is worth mentioning that some users/operators find it more convenient to use the prototyped AR system with the physical touch screen instead of the virtual one, benefiting from buttons on the touch screen and virtual pointers, indicating the connection points, at the AR glasses. Such hybrids, giving a possibility of interactions between the real and virtual worlds, are known as tangible AR (Kato et al., 2000 [48]) and proprioception of those hybrids are understood as a person’s sense of the position and orientation of a person’s body and its several parts (Mine et al., 1997 [49]; Boff et al., 1986 [50]). As an additional explanation, it is worth mentioning that significant and powerful 3D user interface metaphor and convenient 2D desktop metaphor are very important to a user to create a working environment as user-friendly as possible (Schmalstieg et al., 2002 [51]). This is an extremely important argument connected to the fact that the manual wiring production may take the whole working day (considered as 8 h, as one shift of work in the domestic situation of the paper’s authors), therefore a moderation and balance of what users see in front of their eyes for such a long period of time is of the highest importance.

In the production station a user gathers the following elements in order to start a wires assembly process:

- An industrial enclosure (which will form a control cabinet as the result of the production process) with components and wire ducts assembled in the previous production step;
- A set of pre-produced wires, cut to the pre-defined length (optionally, when a wire label reader is available, which is assumed not to be used in this study, wires are marked with an alpha-numeric label which identifies the connection between particular components of a control cabinet);
- A digital CAD project input file for the wiring support system.

**Figure 5:**
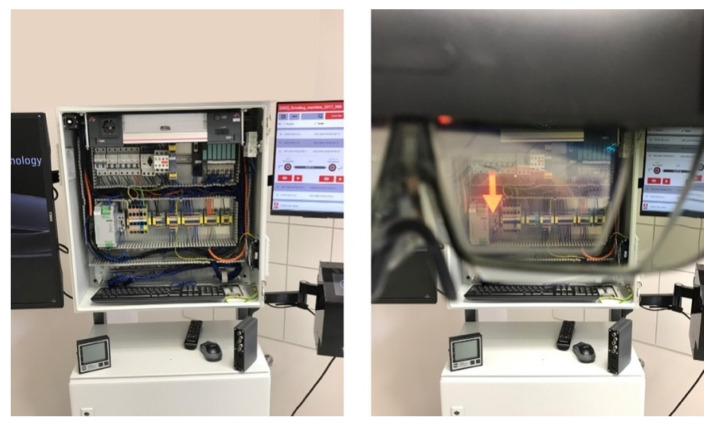
Actual view of a control cabinet; left side: control cabinet seen without the glasses; right side: control cabinet as seen through the right lens of the AR glasses, with virtual red arrow visible (the top grey field is the glasses frame and to the left one can see the glasses bridge and nose pads). Photographs: Szajna A.

The AR glasses enriches the viewed scene with virtual images, i.e., pointers, arrows, and additional images with markings for even better support. The DTPoland AR Smart Wiring 4.0, being a significant part of the station, gives benefits from the interaction between the real and virtual worlds through the application of the aforementioned additional virtual elements, visualized as shown in Figure 4 and Figure 5, directly at the designated area. Figure 4 presents the overview of the production station and the general system principles. It is meant as a graphical illustration of the concept, while Figure 5 presents the actual view of a control cabinet, seen without the AR glasses (left part of Figure 5) and directly through the AR glasses (right part of Figure 5).

The system is designed in order to support the assembly of wires between components in a control cabinet. The system operation process proceeds as follows:

- Step 1: A CAD file is loaded to the wiring support system (ESW as mentioned in Section 2).
- Step 2: A user clicks on the first wire indicated on the list in the ESW system and after that, extended information/data is displayed such as a wire length, connection point details, and 3D graphics for easier navigation The easier navigation can happen either through (a) a virtual screen or (b) a physical touch screen.
- Step 3: A user collects a wire with the appropriate length from a wire holder.
- Step 4: The software related to the AR glasses uses certain coordinates provided by 3D graphics in the ESW, along with particular markers on a cabinet and a matching algorithm, in order to indicate the particular wire’s assembly points through virtual arrows, pointers, virtual images with markings, or textual prompts.
- Step 5: A user indicated with a simple gesture or a voice command on the virtual touch screen that assembly of this particular wire is completed—otherwise, a user is able to report issues to management in real-time and/or create an adequate note. Alternatively, a user can use a physical touch screen to perform these actions.
- Step 6: A user collects the next wire and repeats the operations within the process.

**Figure 6:**
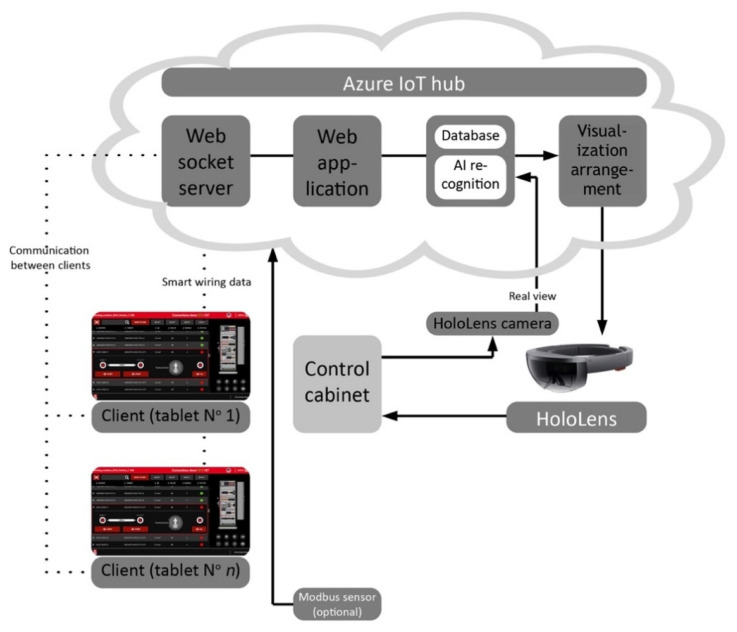
DTPoland AR Smart Wiring 4.0: diagram of the cloud computing-based system with multiple assembly stations, equipped with touch screens and the AR glasses in the wire assembly process of a control cabinet.

The diagram in Figure 6 presents the information flow between the AR glasses, the cloud computing software and the wiring support system (that consists, among others, of web socket server, web application, database, artificial intelligence (AI) recognition subsystem, and visualization module).

**Figure 7:**
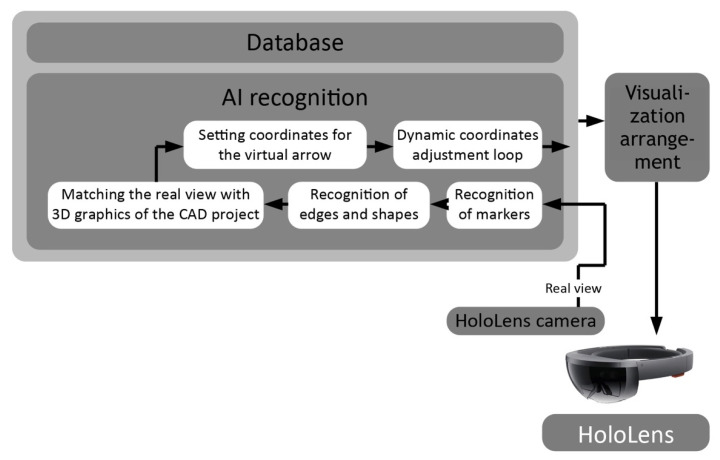
Details of the main DTPoland AR Smart Wiring 4.0 diagram: main functions of the AI recognition subsystem.

Figure 7 presents the detailed part of the AI recognition subsystem diagram given in Figure 6. The main functions of this subsystem are; recognition of markers, edges, and shapes, reflecting and matching of a real-world view with 3D graphics of a CAD scheme as well as setting and adjustment of coordinates for virtual elements (e.g., arrow, dashboard, etc.). The visualization is obviously done by the AR glasses itself. However, the system works in a client-server model (cloud computing technology). The CAD input and data processing, including setting up and arrangement of the visualization (data that in the next step will be simply displayed through the AR glasses) takes place on the cloud computing side. That is why the more accurate term such as “Visualization arrangement” is used in Figure 6 and Figure 7 instead of simply “Visualization”.

One of the biggest challenges of the presented system’s application was the synchronization of the EWS 3D graphics with the corresponding physical industry enclosure/image being seen by the operator. Based on the computer vision tracking techniques, model-based tracking methods were applied to a dynamic coordinates’ adjustment loop. However, based on the situations where CAD data were incomplete or sometimes even misleading, physical markers were essential to enhance this part of the system (Kato and Billinghurst, 1999 [52]). Szajna A. (Szajna et al., 2019 [34]) effectuates the development of the software, which synchronizes the ESW 3D graphics with the image being seen in the AR glasses, towards the elimination of physical markers and application of recent methods in machine learning, precisely, feature learning and deep learning (Quoc et al., 2012 [53]), representing the breakthrough approach in AI (Hinton et al., 2006 [54]; Bengio and LeCun, 2007 [55]). The creation of two-dimensional and three-dimensional representations displayed by the AR glasses used in the presented research, is a fusion of Unity (a cross-platform software engine developed by Unity Technologies), open-source modules and libraries, merged with the proprietary software aided by deep learning technology. Szajna A. (Szajna et al., 2019 [34]) expects to provide the ability of the system to automatically recognize a whole control cabinet and its individual components, viewed directly through the AR glasses in the actual environment. Such improvement is expected to allow the elimination of one of the steps from the control cabinet production operation, namely the need to attach physical markers to every single control cabinet.

## 4. Production Process Study: Methodology, Test Setup, and Testing Procedure

A key activity in developing AR experiences is evaluating the usability of the AR application (Billinghurst et al., 2015 [56]). The aim of this study was to show the difference between using the manual wiring support system with and without the AR support application. The only distinction in the test procedure was the use of the Microsoft HoloLens smart glasses, giving the 3D information right in front of a user’s eyes, instead of the need of interpretation by the 3D graphics on the touch screen and matching it with the actual control cabinet. The HoloLens is equipped with powerful computer chipsets and a state-of-the-art display with the user’s field of view wider than in other devices of such types e.g., Google Glass and Epson according to Lee and Hui (2018) [27]. This device consists of following sensors and components, such as a camera, a microphone, a GPS, an accelerometer, a gyroscope, a magnetometer, a light sensor, and an optical display. The chipsets in these AR glasses create a more user-friendly environment, which allows the user to pin holograms onto the surrounding physical environment (Lee and Hui, 2018 [27]), which is a significant feature to facilitate during the control cabinets assembly.

The presented solution was tested under a particular methodology, which is defined in the following subsections.

### 4.1. Production Process Study: Methodology

The authors carried out an empirical study based on the experimental method. The examined process was isolated from disturbances/side effects, i.e., uncontrolled factors like lack of components or wires, wrongly assembled elements, or errors in the CAD design. The conditions of experiments were ideal in order to focus only on assembly duration both with and without the AR support system. The single one significant variable was the employment of the AR glasses used for transmitting particular information presented in the right place in front of the user’s eyes.

The following experimental method’s steps were specified and described during the conceptual phase of the actual study:

- Step 1: Observation. The operator commences with the wiring process, aided by the touchscreen support system (ESW), looks at the screen with the 3D graphics, and makes interpretations (zooming in and out as shown in Figure 3); a repetitive activity that takes time.
- Step 2: Hypothesis. The convenient human-machine interface, based on the AR support system, should allow a user to save time on performing the manual wiring production process, without causing meaningful discomfort or difficulties in performing the task, in comparison to performing the same tasks in the process without using such a support system.
- Step 3: Prediction/assumption. If a task duration comparison is made between completing the task with the aid of the support system and without the AR glasses, the use of the AR support system should result in a shorter duration.
- Step 4: Experiment specified in Table 1.
- Step 5: Result analysis, synthesis, and conclusions.

### 4.2. Production Process Study: Arrangement and Configuration

Particular arrangements and configuration of the test station are given in the current subsection. Final preparations of the test station, right before the experiment, consisted of the steps given in Section 4.4.

The test station with the prototyped AR support system given in Figure 4 consisted of devices and equipment, listed below:

- An industrial enclosure (which forms a control cabinet after components assembly) with:
  - preliminary assembled components on DIN rails which is a metal rail of a standardized type (according to DIN norms), widely used for mounting circuit breakers and industrial control equipment inside equipment racks/enclosures (these components can be elements requiring a screwdriver to screw a wire into it or push-in without screwing),
  - preliminary assembled wire ducts;
- 4 sets of 20 wires each prepared in advance, with CAD system pre-defined lengths: 20 cm, 50 cm, 70 cm, 110 cm, 150 cm, 200 cm, 250 cm, and 300 cm;
- A wire holder dividing each wire length type in a separate position;
- A manual wiring support system: EPLAN Smart Wiring (ESW);
- The AR glasses (Microsoft HoloLens), used for testing “with glasses” (50% of the overall test was conducted with the aid of AR glasses, whereas in order to compare the durations of tasks in the process, another 50% of tests were arranged without any AR glasses).

### 4.3. Assumptions and Conditions of the Tests

In order to make the tests results more representative and factual (with reference to the actual work during control cabinets production), the research team performed the tests with the following varied assumptions and conditions. The tests were proceeded in two different locations of test sites: the Rittal Innovation Centre (Haiger city, Germany) and the DT Poland Research Centre (Zielona Gora city, Poland). In order to perform the tests with the variant equipment, four types of industrial enclosures (which form a control cabinet after components assembly) were introduced with the following quantities: 1 unit of Rittal VX25, 1 unit of Rittal TS8, 2 units of Rittal AE, with 4 different CAD schemes. The industrial enclosures were assembled with the use of 4 different sets of wires, 20 pieces each, with 8 different CAD system defined lengths (the wire configurations were the same, however with different mounting positions within the cabinet).

Different types of industrial enclosures and various sets of wires were applied with the intention to reduce the learning effect between consecutive tasks.

The tests were performed according to the particular procedure described in Section 4.4.

### 4.4. The Test Procedure

The test procedure performed on the test station with the prototyped AR support system given in Figure 4. It consisted of preparation steps and accordingly the measurement steps are mentioned separately. Preparations steps were, as listed below:

- Step 1: Providing a pre-assembled industrial enclosure (which forms a control cabinet after components assembly) to the test station and fixing it on the assembly frame.
- Step 2: Collecting all 20 wires in the right slots of the wire holder.
- Step 3: Preparing screwdrivers for certain operations.
- Step 4: Activating a manual wiring support system on a touch screen and loading the CAD file.
- Step 5: Activating the AR glasses during the tests ‘with AR glasses’ being used.
- Step 6: Preparing a stopwatch.

Another part of the test procedure was the following measurement steps:

- Step 1: Running the stopwatch.
- Step 2: Repeating the manual wiring cycle 20 times (for 20 wires separately), as is it given in Table 1.
- Step 3: Stopping the stopwatch after the twentieth wire assembly in an industrial enclosure.
- Step 4: Writing down the stopwatch indications.

It is important to define what is considered to be a success and what would be understood as a failure during the tests. Success in a control cabinet production means a full assembly of a unit. An assembly with the AR glasses taking longer than assembly without the AR glasses is also considered a failure.

The group of testers consisted of two operators, both possessing higher technical education and heterogeneous characteristics:

- Tester 1: male born in 1983, has worked for 6 years in the industry (including a position directly connected to control cabinet assembly);
- Tester 2: male born in 1989, has worked for 3 years in the industry (including a position directly connected to control cabinet assembly).

It is worth mentioning that the system’s operators are given visual indications on which connector is the right one through the visualization either on the ESW support system or directly through the AR glasses.

The test procedure implied an important benchmarking research question of the study, mentioned in the introduction to this paper. Therefore, the authors suggested this particular benchmarking research to be analyzed. The benchmarking research question reads as follows: *How long does it take for a person to look at a touch screen, interpret 3D graphics on a wiring support system (perform the 4-step usage of the ESW, described in Figure 3) and match it with an actual industry enclosure* vs. *how long does it take for a person to see all this information through the AR glasses right in front of their eyes indicating the correct wire connection point?* To paraphrase this benchmarking research question in brief: what are the differences in the full process while points 3, 6, and 8 specified in Table 1 are varied? The answers to these questions are given in the next section.

## 5. Production Process Study: Results of the Prototype Tests, Performed in the Actual Production Environment

**Figure 8:**
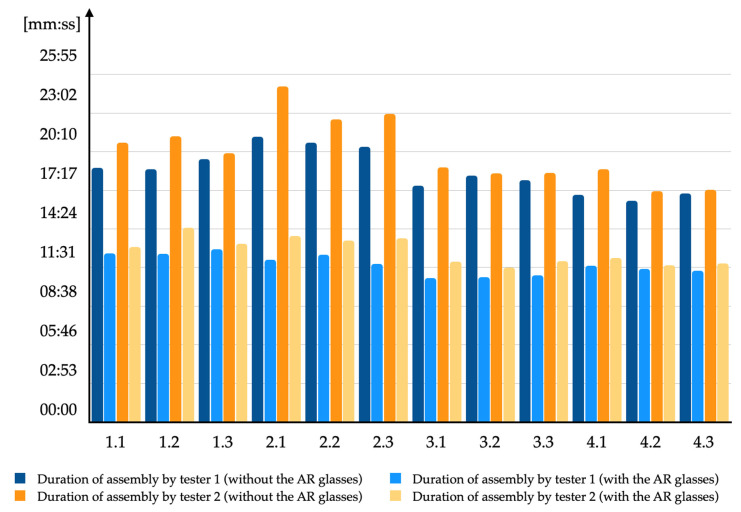
Measured values and calculations from all 48 tests.

The prototype tests were performed in the actual production environment according to the procedure defined in the previous section. The whole study was based on 48 individual tests. Each tester performed 24 individual tests. For four different industrial enclosures, 6 tests per one person were arranged with the application of each industrial enclosure (3 tests with the AR glasses and another 3 without them). The tests #1.x and #3.x were performed with the use of industrial enclosures Rittal VX25 and Rittal AE, whereas the tests #2.x and #4.x were performed with the use of Rittal TS8 and Rittal AE, as given in Table 2 and Figure 8 (without # sign). Test signatures, such as #1.x, connected to the symbols on the test number follow the symbols presented in Table 2. It should be mentioned that industrial enclosure assembly frames and wire holders slightly varied between these two locations.

It should be also mentioned that previous to the tests described in the paper, numerous preliminary tests had been conducted, however, those were connected with the development of the system in order to make it a ready-made system for the tests described in the paper’s study. Preliminary tests are not mentioned in this paper. 

Table 2 presents the measured values of control cabinet production’s duration and adequate calculations for all predefined tests. Each time, setting up and configuration of the system have been executed, in addition, preliminary tests were conducted as well. As a rule, one test took approximately an hour and during that time, all the test procedure activities were performed.

## 6. Results Discussion

In Table 2, based on the conducted tests, it is explicit that the use of the AR glasses saves time in the examined case. The operator/tester not wearing the AR glasses, who looks at the screen with a wiring support system diagram and interprets the 3D graphics (with a question in mind: “where is the actual 3D wire connection point?”) requires time that could be saved with the use of the AR system’s prototype.

In the tested process of a 20-wire assembly, the minimum saved time was 4:48 [mm:ss]. The complete assembly process without wearing the AR glasses, took 15:35 [mm:ss], therefore the production process time decreased by 31%. Meanwhile, the maximum time saved was 10:32 [mm:ss]. The complete process without wearing the AR glasses took 23:38 [mm:ss]. In this test, the production process time decreased by a surprisingly high value which was 45%. Approximately 6.5 to 7.0 min were saved on average. That gave an average from 36 to 37% decrease in the time needed to perform the manual assembly process. Taking into consideration the fact that a fully equipped control cabinet consists of not 20 wires, but considerably more (from 150 to 300 wires), time savings of around 30–35% are noticeable and substantially colossal, especially that they imply compelling financial savings. The CTO of the industry giant Rittal GmbH & Co. KG, Steffen Thomas, mentioned that savings up to 2% in the industry are considered desirable, meanwhile saving on the level between 30 and 40% makes a breakthrough (2017, personal communication). In order to substantiate this statement, it is worth noting the aviation giant Boeing implemented a 25% improvement in production time (25% of time-saving) and achieved a higher first-time quality in their wire assembly, Duin et al. (2019) [57]. High average usability scores were also confirmed in independent research connected to cooperation between pairs of the AR glasses (of the same type as in this study) by the research of Sluganovic et al. (2017) [58].

Average values are the type of statistics that might be interesting as a result obtained in these tests, however, it is of particular interest to compute the strength of correlation between certain sets of values. In order to compute the strength of the correlation, Pearson’s correlation coefficient was used. 

To determine the correlation strength, the following criteria were identified and interpreted (according to Guilford’s interpretation of the magnitude of significant correlations, Tredoux and Durheim, 2004 [59] after van Aswegen and Engelbrecht, 2009 [60]):

- slight, almost no relationship, |*r_xy_*| < 0.19;
- low correlation, definite but small relationship, 0.20 ≤ |*r_xy_*| < 0.39;
- practically significant relationship |*r_xy_*| ≥ 0.30;
- moderate correlation; substantial relationship, 0.40 ≤ |*r_xy_*| < 0.69;
- high correlation; strong relationship, 0.70 ≤ |*r_xy_*| < 0.89, 0.70–0.89;
- very high correlation very dependable relationship, 0.90 ≤ |*r_xy_*| < 1.00.

The results connected to the strength of correlation for the particular sets of data are described below. The strength of correlation between the values measured in the case of tester 1 without wearing the AR glasses during the tests (noted as *t1ØAR*) and tester 1 wearing the AR glasses during the tests (noted as *t1AR*) was equal to *r*(*t1ØAR*; *t1AR*) = 0.572814344 (moderate correlation) with a probability of a type I error determined as *p* = 0.052. This value of correlation coefficient refers to the relation which depends on the fact that the use of AR glasses by tester 1 was very beneficial during the control cabinet assembly process in comparison to not using the AR glasses. This advantage was connected to a significant reduction in the duration of individual operations and consequently, the entire control cabinet assembly process. The reduction in duration is indicated in Table 1 in the column described “time duration decreased by”. When the value of the correlation coefficient was changed to a qualitative term, it was found that it is a moderate correlation. This means that there are certain correlations between the process duration values for individual tests both with and without using the AR glasses; certainly, it could not be otherwise, since the arrangement, assumptions, and plan of the test course with and without the AR glasses was identical. Moderate correlation in this case also proves the fact that tester 1 showed quick recognition (fast learning) of the devices (industrial enclosures) on which he was working, so the times of individual operations in cases of tests without and with using the AR glasses could be comparable in terms of values. The faster the recognition of the equipment, the shorter the duration of processes with the AR glasses. It is worth noting that the device recognition acumen is an individual feature of the person who realizes the process.

Meanwhile, the strength of correlation between the values measured in the case of tester 2 not wearing the AR glasses during the tests (noted as *t2ØAR*) and tester 2 wearing the AR glasses during the tests (noted as *t2AR*) was equal to *r(t2ØAR; t2AR)* = 0.847575454 (high correlation) with a probability of a type I error determined as *p* = 0.001. The results of this correlation coefficient calculation mean that tester 2 may have had slower recognition of the devices (tester 2 is younger and had less experience in the industry). On the one hand, the coefficient value was determined based on longer process times per test. On the other hand, the value of the coefficient expressed as a qualitative expression indicates a high correlation. Another reason for that the quickness of device recognition is an individual feature of the operator performing the process. It is worth noting at this point that this feature had no significant impact on the overall result of all tests, namely that the decrease of time needed to perform the manual wiring process was between 36 and 37% on average.

The strength of correlation between the values measured in the case of tester 1 and tester 2 wearing the AR glasses during the tests (noted adequately as *t1AR*, *t2AR*) was equal to *r*(*t1AR*; *t2AR*) = 0.803832588 (high correlation) with a probability of a type I error determined as *p* = 0.002. The results of this correlation coefficient calculation mean that the effects of the conducted research are convergent with each other due to the high similarity between the values of the duration of the control cabinet assembly process carried out with aid of the AR glasses by two various testers. Moreover, there were no significant deviations between the course of the solution testing with the use of the AR glasses by tester 1 and tester 2.

The strength of correlation between the values measured in the case of tester 1 and tester 2 not wearing the AR glasses during the tests (noted adequately as *t1ØAR*, *t2ØAR*) was equal to *r* (*t1ØAR*; *t2ØAR*) = 0.932270421 (very high correlation) with a probability of a type I error determined as *p* < 0.001. The higher correlation coefficient’s value is related to the fact that the process of control cabinet assembly without the use of the AR glasses was carried out according to classic, and therefore familiar procedures to testers. In future analyses, it can be expected that the correlation coefficient’s values of the *(t1AR; t2AR)* type will be described as a very high correlation.

Another correlation coefficient value might be of interest to researchers. This value signifies the correlation between time saved in the process performed by tester 1 (noted as *t1_s*) and the same for tester 2 (noted as *t2_s*) was equal to *r* (*t1_s*; *t2_s*) = 0.883008194 (high correlation) with a probability of a type I error determined as *p* < 0.001. The results are very promising, and it is worth mentioning that the results of the last correlation mean that the application of the prototype in the actual production of control cabinets would assure high timesaving regardless of the operator’s features. Certainly, the time saving is very promising when average values are compared (from 36 to 37% of time-saving in the process is a significantly acclaimed value; it is a significant contrast in relation to the hypothesis that a user saves time on performing the manual wiring production process with the use of the AR glasses in comparison to the process without the use of the AR glasses, not causing meaningful discomfort or difficulties in performing the task).

The values obtained during the repeated tests matched samples in pairs, for example (*t1ØAR*; *t1AR*) as it was presented earlier in this section. The other possibility to assess whether values sampled differ, in the same pairs as for correlation coefficient calculations, is the Wilcoxon signed-rank test. It is a non-parametric statistical hypothesis test used to compare two related samples. 

The null hypothesis is defined as follows: the convenient human-machine interface, based on the AR support system, allows a user to save time on performing the manual wiring production process, without causing meaningful discomfort or difficulties in performing the task, in comparison to performing the same process tasks without using such support system. 

The alternative hypothesis is defined as follows: the convenient human-machine interface, based on the AR support system, does not allow a user to save time on performing the manual wiring production process, without causing meaningful discomfort or difficulties in performing the task, in comparison to performing the same process tasks without using such a support system. 

It has to be mentioned that for all the pairs except one, the rankings used in the Wilcoxon test were on either side of statistics *T_−_* or *T_+_*. The only pair of time saved values (*t1_s*; *t2_s*) is different from the other pairs that both *T_−_* and *T_+_* are not equal to zero. This is why the definition of other hypotheses and corresponding results for the other pairs, but the aforementioned ones, are not presented. In the case of this pair, *T_−_* (*t1_s*; *t2_s*) = 62.5 and *T_+_* (*t1_s*; *t2_s*) = 15.5, therefore the critical value of statistics is *T* = 15.5. When assuming the significance level equal to α = 0.1 then the region of acceptance is [0;17]. In this case, the statistic is found in this region so that the null hypothesis can be accepted with a probability of a type I error determined as *p* = 0.0002. Meanwhile, assuming the significance level equal to α = 0.05, the region of acceptance is [0;13]. In this case, the statistic is not found in this region, as opposed to the previous case, the null hypothesis cannot be accepted, and the alternative hypothesis may be accepted with a probability of a type I error determined as *p* = 0.00024.

The value of α is conventional. Usually α = 0.05, α = 0.01 or α = 0.001 are assumed. This makes the same statistical hypothesis likely to be relevant with à priori a higher value of α and not relevant to a lower one. On the basis of this general statement and the obtained quantitative results, the authors concluded that the application of the correlation coefficient was more authoritative in this research.

The research was conducted during the first shift. Testers performed the runs alternately (first tester 1, then tester 2), which means after completing the single exercise, the tester could take a rest (c.a. 15–20 min) so that fatigue is not a parameter that significantly affects the test operation’s results. Extended testing in future research may have an approximate plan, as follows: tests before and after a meal, and before the end of the work, so that the fatigue parameter could be taken into consideration and investigated as to how it affects the test results.

During test arrangements with the aid of the AR support system, both variations mentioned in Section 3 (Step 2), namely with (a) the virtual screen and (b) the additional physical touch screen were practiced before the final tests. The difference between the two variations was in displaying additional information and the way of clicking the status button after finishing each wire assembly (touching the physical button vs. making a gesture to click the virtual one). This was partly recorded in the study, however, it seemed not to influence the production process duration.

The screen was placed next to the particular control cabinet, on its right side. This placement was extremely convenient when working with smaller enclosures such as Rittal AE. In the case of 2-m high enclosures such as Rittal TS8 and VX25, it was complicated for the testers, who had to squat during the tests’ performance. On the other hand, the physical wire holder placed below the screen caused additional movements for the testers. It was possible to easily move the virtual screen, but the initial limitation to 20 wires made testers not do it; it was constant for all the test runs so that no additional variable occurred. For the large, fully equipped control cabinet consisting of not 20, but circa 150–300 wires, this functionality could prove to be useful.

It is worth stressing that all the tests were finished successfully which means that all the control cabinets were fully assembled. During the tests, problematic situations such as the lack of certain wires, wrongly assembled elements or an error in the CAD design, which would result in the impossibility of assembling a cabinet (failure), have not occurred. The conditions were ideal in order to allow the testers to focus solely on the assembly while working both with and without the AR glasses support. Each assembly was a success, at the time it was recorded. There was no assumption that assembling over 25 min is a failure. 

Although the study scope was planned to give reliable results by introducing variants in the form of 4 cabinet types and 4 CAD schemes, 2 different locations, 2 testing people and 48 test runs, the single test run was limited to only 20 wires, which potentially could evoke inaccuracies. However, the use of a limited number of 20 wires was acceptable at that stage, since, to the authors’ best knowledge, there is no other available research on the AR glasses in the actual production environment and the actual production process of manual wiring. 

From the study described in the paper, it is apparent that the AR glasses can have a significant impact on improving: (a) selected performance indicators, (b) ergonomics (e.g., work convenience, relieving from learning by heart, browsing through documentation, availability, and presentation of information), and (c) organizational and management matters (e.g., gathering and managing information, shortening response time to an inquiry regarding the searched information).

The study was based on a single, specific production process; however, the authors confirmed an expectation about the universality of using AR technology in manual process activities in terms of improving selected performance indicators. Since AR technology delivers appropriate information (accurate, useful, and up-to-date), in the right place at the right time, AR seems to be an ideal solution leading to shorter production times, less training effort, reduction of errors, and finally reducing production costs (Haller et al., 2006) [61].

## 7. Technology Imperfections, Limitations, and Future Development

In the study, it was important to use market available hardware, since it may ensure faster readiness to introduce the proposed solution on a bigger scale. Therefore, it should be mentioned that three other types of AR glasses were tested, as during preliminary tests, namely Meta 2, Epson Moverio BT-200 (used e.g., by Angrisani et al., 2018 [22]), Epson Moverio BT-300, and the Google Glass. Only HoloLens was chosen for the in-depth study (used e.g., by Ingeson et al., 2019 [15]; Müller et al., 2018 [17]; Kot et al., 2018b [24]; Vorraber et al., 2019 [28]; Sluganovic et al., 2017 [58]; Xue et al., 2019 [62]) and presented the most advanced technology with the widest vision field and 3D space, giving the most user-friendly interface, especially in accordance with the assumptions made in this research. Because of the assumptions mentioned above, the AR support system was not adapted and installed on other models of AR glasses. 

As the selected authors of this paper work for a commercial company, they made preliminary analysis and as a result, they decided to choose the best possible equipment to meet the demands of the research planned in advance. Tests comparing HoloLens to other types of AR glasses were not carried out during the research. Nevertheless, the mentioned preliminary analysis made it possible to exclude other AR glasses equipment out of concern for the competition rules, various remarks connected to the use of other companies’ glasses, without indication of their names, are mentioned below.

One of the significant aspects of the project was the possibility to overlay 3D graphics on the actual image. Some of the other AR glasses are equipped with a considerably larger screen, however, their ambient or depth recognition support was not satisfying. On the other hand, some other AR glasses are equipped with too small screens, in case of which a display field for virtual elements was located on the edge of the display. This might have caused relatively early user fatigue due to the lack of an actual overlay of the virtual elements right in front of the user’s eyes. Other reasons were, to mention only the most important from the perspective of the system’s inventors: lack of software support, lack of mobility (wired AR glasses), lack of ambient scanning sensors, screening calibration not compatible for another person but the particular user, lack of 3D holograms display, the problem of fixed eyes’ pupils spacing (some users see well through AR glasses and some other do not—they might get tired relatively fast), poor quality of spatiality (users could anchor holograms, nevertheless such action takes a lot of effort during equipment’s calibration), there is no gesture control (e.g., hand movement) but a manipulator, etc. It is worth mentioning that other AR glasses, to the one used in this study, may fit better for other use cases, especially when it is not necessary to overlay the 3D elements in the right place of the real environment.

The system described in the paper requires continuous study. In the desired highest quality system for control cabinet production, precision suffers from the low-specialized AR components used in the hardware, posing one of the biggest disadvantages for the present moment. It is fairly difficult to coordinate the 3D screening of the exact point, keeping it directly at a pre-defined spot while the user’s head is moving, when approximately 0.5 mm precision is required. Even the advanced software concepts for the adaptative algorithm presented unsatisfactory results. Moreover, the software cannot always overcome hardware issues. The observed system’s tests showed that the more software for improving the precision was cumulated, the longer the delay in the real-time perception occurred. Achieving low latency tracking with high precision and accuracy is still challenging (Kim et al., 2018 [30]).

In the research team’s opinion, the HoloLens ver. 1 construction is not reaching industrial requirements. Especially weight, precision, and fragility of construction are the main issues. In one case, the device’s band broke, making it impossible to keep the AR glasses on a tester’s head, and its use was far from demanding lengthy production. Battery working time was shorter than expected, however, it could be extended by the use of a wired power bank, which proved to be quite a convenient solution. It was used during hours of continuous research tests. Certainly, some use cases find HoloLens ver. 1 suitable.

Minor inconveniences to eyesight were detected after longer use of the analyzed AR glasses. This topic however requires longer and deeper research to formulate theories. It is certain that the AR gives the user more of an experience that is perceived as more natural in comparison to virtual reality. Obviously, the AR is far from some virtual reality issues for human senses’ experience, considering it positively for AR technology. At the time the authors did not find more extensive research on this topic. Some aspects were found in the literature e.g., Flavián et al., 2019 [63]); however, such a state of matters is not surprising for a tool that has not been available long enough on the world market sales.

More limitations were observed during the parallel research on the production line monitoring. The authors intended to describe the outcome of the abovementioned research in detail in a forthcoming paper.

The authors believe, that expert testers should be engaged in the system efficiency, performance, and added value tests on a wider scope. On the one hand, two testers might not be sufficient for the system’s testing, on the other hand, during the research, they executed a total of 48 individual tests, which was a remarkable value when high expenditures of the prototype and high expenses of the testing phase were incurred. Apart from the expenditures and other financial costs, the time consuming preliminary and final tests were the other significant reason for the number of testers and tests conducted during the research. The time consumption, expenses, and complicated organization of technical matters caused by the necessity to introduce numerous preliminary settings (IT issues are not a subject of this paper), and, at the same time, press reports on a new version of the tested AR glasses (White, 2019 [64]) prompted the author’s decision to finish the tests at this stage and resume them when the new version of the AR glasses is available. It is worth taking into account that developers of the AR glasses are constantly upgrading their products, therefore the HoloLens software state during the period of May 2019–May 2020, when the research was conducted, may undergo considerable changes in the course of oncoming months, concerning the pace of the development and demand for research in the field of AR. HoloLens ver. 2 was presented during the Mobile World Congress in Barcelona (February 2019; White, 2019 [64]). The second version of the AR glasses was available for the preliminary order with no specified shipment date during the time the research described in this article was conducted. Nevertheless, the authors are eagerly waiting to start tests of the described system with the implementation of HoloLens ver. 2. The authors plan to resume the same test procedure described in this article as soon as they receive the 2nd version of the AR glasses and compare the results of both studies. Additionally, the authors plan to carry out long-term tests, employing the AR system for several hours, which would simulate an ordinary workday.

A further development and enrichment of the AR wiring system are known to lead towards new functionalities for different persons in the value chain, such as a) testing of a control cabinets’ wiring or b) servicing cabinets, having meaningful information automatically at hand, in the form of a cloud computing-based support system consisting of e.g., an automatic meaningful event register and service book. Such a system would, fully or at least partially, connect control cabinets to reality (Azuma, 2016 [65]), so when the AR glasses detect any of the control cabinets in any geographical location in the end-user’s premises, it will automatically recognize the unit and provide the user with appropriate data. Additionally, apart from that the functions mentioned above, as mentioned in Section 3, recognition and synchronization software is planned to be enriched with AI. The authors plan to follow the development of the system with a corresponding evaluation.

## 8. Conclusions

More and more studies connected to the AR area of Industry 4.0 in the actual production environment appear in the scientific literature. One study on the topic of applied AR—AR solution, which reduces downtime in the event of a malfunction—was lately described by researchers from the Graz University of Technology (Viehberger et al., 2019 [66]). Also, at the University of Zielona Góra, the research team investigates AI and AR possibilities in the process of production maintenance in the Industry 4.0 context (Szajna et al., 2018 [67]). Apart from the AR application for manual wiring support in the control cabinet production process, Szajna A. along with a research team is working on another significant topic concerning Production 4.0: Deep Neural Network supported computer vision for the intelligent robotic arm, giving the robot freedom in making decisions, based on an observed situation.

The aim of the research presented in the paper was to describe the most recently developed system for wire assembly and production process and particularly present tests of the system’s prototype as an assistance device for the wiring of control cabinets. The system works on-line, identifying the elements of the environment and designating significant support to a user (a particular operator in control cabinets’ assembly and production line). The software has been validated by two testers, experts in the analyzed industry sector, through a large number of tests. The tests proved that the system equipped with the AR glasses supports the process of control cabinets production and therefore, the answer for the benchmarking research question of the study was indicated. The question is as follows: *How long does it take for a person to look at a touch screen, interpret 3D graphics on a wiring support system (perform the 4-step usage of the ESW, described in Figure 3) and match it with an actual industry enclosure* vs. *how long does it take for a person to see all this information through the AR glasses right in front of their eyes indicating the correct wire connection point?* A significant objective has been achieved in the presented study. As a result of the research and carried out tests, it has been demonstrated that the wire assembly process can be significantly shortened due to the use of the prototype system with the AR glasses support presented in the article. The full answer to the benchmarking research question is presented in detail in Section 4, Section 5 and Section 6 of the paper. The authors declare that the described research can enter the next phase of testing and subsequent plan of implementation on the global market.

Thanks to that solution, it has become possible to introduce students and young employees, during their first practice, to the actual working environment as employees of large companies with committed safety programs in place, as suggested by Evans (2013) [68] and analyzed by Khan et al. (2019) [69].

The development of the analyses will be based on the study of Xue et al. (2019) [62] that established a set of norms for the evaluation of user satisfaction of using AR glasses and AR applications. Moreover, most of the studies are focused on ergonomic analysis, meanwhile, the research on the aesthetics of interface layout was insufficient as it was claimed in Deng and Wang (2020) [70]. This is one of the potentials for the future development of the described solution. Especially as one of AR’s most recognized experts, Azuma R.T. stated recently that there still is much research and development which remains to achieve the vision of ubiquitous, socially acceptable AR systems, Azuma (2019) [71]. This topic presents promising possibilities for the future development of the described solution.

## Figures and Tables

**Table 1:** Operations during step (2) of the test procedure (optional operations in the case of the test without any AR glasses or with the AR glasses are marked as ‘a’ or ‘b’ next to the number or particular operation).

No.	Operation Description	Operation Without any AR Glasses	Operation with the AR Glasses
1	Opening a wire’s extended information in the ESW system, read a wire’s length	✓	✓
2	Taking a wire of the correct length from the holder	✓	✓
3a	Interpreting the graphics on the ESW system in order to assess where to assemble the first ending in Position A	✓	×
3b	Observing in the AR glasses, right in front of one’s eyes, at the right spot of a physical industrial enclosure, where to assemble the first wiring ending in Position A	×	✓
4	Assembly of the first wire ending in Position A (it can be screwed secured by a screw or pushed-in)	✓	✓
5	Leading a wire through wire duct fingers into proximity of Position A	✓	✓
6a	Interpreting graphics on the ESW system in order to know how to lead a wire through the wire duct towards Position B	✓	×
6b	Observing through the AR glasses, how to lead a wire through a wire duct towards Position B	×	✓
7	Leading a wire through a wire duct (placing a wire between Position A and B)	✓	✓
8a	Interpreting the graphics on the ESW system in order to know where to assemble the second wire end in Position B	✓	×
8b	Observing through the AR glasses, where to assemble the second end in Position B	×	✓
9	Assembly of the second wire end in Position B (it can be secured by a screw or pushed-in)	✓	✓
10	Leading a wire through wire duct fingers into proximity of Position B (depending on the tester preferences and conditions, this point could take place here or in the previous step—before the assembly of the second wire end in Position B)	✓	✓

**Table 2:** Measured values and calculations from all 48 tests (colors given at the bottom part of this table are connected to appropriate columns’ colors given in Figure 8).

No.	Industrial Enclosure Used for the Test	Test #	Tester 1 without the AR Glasses [mm:ss]	Tester 1 with the AR Glasses [mm:ss]	Time Saved [mm:ss]	Time Duration Decrease by: [%]	Tester 2 without the AR Glasses [mm:ss]	Tester 2 with the AR Glasses [mm:ss]	Time Saved [mm:ss]	Time Duration Decrease by: [%]
1	Rittal VX25	1.1	17:54	11:53	06:01	34%	19:41	12:19	07:22	37%
		1.2	17:49	11:51	05:58	33%	20:07	13:41	06:26	32%
		1.3	18:31	12:10	06:21	34%	18:55	12:33	06:22	34%
2	Rittal TS8	2.1	20:06	11:25	08:41	43%	23:38	13:06	10:32	45%
		2.2	19:41	11:47	07:54	40%	21:18	12:47	08:31	40%
		2.3	19:22	11:09	08:13	42%	21:41	12:56	08:45	40%
3	Rittal AE	3.1	16:38	10:08	06:30	39%	17:55	11:17	06:38	37%
		3.2	17:21	10:12	07:09	41%	17:31	10:52	06:39	38%
		3.3	17:01	10:19	06:42	39%	17:33	11:19	06:14	36%
4	Rittal AE	4.1	15:59	11:01	04:58	31%	17:48	11:33	06:15	35%
		4.2	15:35	10:47	04:48	31%	16:15	11:02	05:13	32%
		4.3	16:06	10:39	05:27	34%	16:22	11:11	05:11	32%
										
			Average value for tester 1:		06:34	37%	Average value for tester 2:		07:01	36%

## References

[1] Navaei J., ElMaraghy H. (2018). Optimal operations sequence retrieval from master operations sequence for part/product families. Int. J. Prod. Res..

[2] Syberfeldt A., Holm M., Danielsson O., Wang L., Brewster R.L. (2016). Support systems on the industrial shop-floors of the future—Operators’ perspective on augmented reality. Procedia CIRP.

[3] Gilchrist A. (2016). Industry 4.0: The Industrial Internet of Things.

[4] Gorecky D., Schmitt M., Loskyll M., Zühlke D. Human-machine-interaction in the industry 4.0 era. Proceedings of the Industrial Informatics (INDIN) 12th IEEE International Conference.

[5] Zeng M., Guo G., Tang Q. Vehicle human-machine interaction interface evaluation method based on eye movement and finger tracking technology. Proceedings of the 21st International Conference on Human-Computer Interaction, HCII 2019: HCI International 2019—Late Breaking Papers.

[6] Zhao L., Zhang L., Wang Z., Tian G., Zhang L. (2018). Design of human-machine interaction interface with multiple views for dispatching automation system. Autom. Electr. Power Syst..

[7] Zhang B., Ding M., Li Y. (2016). Optimization design of human machine interaction interface based on visual perception. Zhongguo Jixie Gongcheng, China Mech. Eng..

[8] Deng L., Wang G., Yu S. (2016). Layout design of human-machine interaction interface of cabin based on cognitive ergonomics and GA-ACA. Comput. Intell. Neurosci..

[9] Azuma R.T. (1997). A survey of augmented reality. Presence Teleoperation Virtual Environ..

[10] Pereira A.C., Arezes P., Alves A.C., Duarte F.J. An enhanced human-machine interface enabled by augmented reality—A new approach for human augmentation. Proceedings of the XIX International Conference on Occupational Risk Prevention.

[11] Ro Y.K., Brem A., Rauschnabel P.A., Jung T., tom Dieck M. (2018). Augmented reality smart glasses: Definition, concepts and impact on firm value creation. Augmented Reality and Virtual Reality.

[12] Cutolo F., Mamone V., Carbonaro N., Ferrari V., Tognetti A. (2020). Ambiguity-free optical—Inertial tracking for augmented reality headsets. Sensors.

[13] Choi J., Park H., Paek J., Balan R.K., Ko J. LpGL: Low-power graphics library for mobile AR headsets. Proceedings of the 17th Annual International Conference on Mobile Systems, Applications, and Services, MobiSys 2019.

[14] Li Z., Mo X., Shi C., Jiang S., Jiang L., Ahram T. (2020). The research of visual characteristics for the deaf wearing AR glasses. Advances in Intelligent Systems and Computing, Proceedings of the Advances in Human Factors in Wearable Technologies and Game Design, AHFE 24–28 July 2019.

[15] Ingeson M., Blusi M., Nieves J.C. (2019). Microsoft Hololens—A mHealth solution for medication adherence. Lecture Notes in Computer Science (including subseries Lecture Notes in Artificial Intelligence and Lecture Notes in Bioinformatics), Proceedings of the 1st International Workshop on Artificial Intelligence in Health, AIH 2018, Stockholm, Sweden, 13–14 July 2018.

[16] Ong S.C., Pek I.L.C., Chiang C.T.L., Soon H.W., Chua K.C., Sassman C., Koh V.T.C. (2019). A novel automated visual acuity test using a portable augmented reality headset. Investig. Ophthalmol. Vis. Sci..

[17] Müller C., Krone M., Huber M., Biener V., Herr D., Koch S., Reina G., Weiskopf D., Ertl T. (2018). Interactive molecular graphics for augmented reality using hololens. J. Integr. Bioinform..

[18] Zheng M., Campbell A.G. Location-based augmented reality in-situ visualization applied for agricultural fieldwork navigation. Proceedings of the 18th IEEE International Symposium on Mixed and Augmented Reality, ISMAR-Adjunct 2019.

[19] Angrisani L., Arpaia P., Esposito A., Moccaldi A. (2020). A wearable brain-computer interface instrument for augmented reality-based inspection in industry 4.0. IEEE Trans. Instrum. Meas..

[20] Govindarajan U.H., Trappey A.J.C., Trappey C.V. (2018). Immersive technology for human-centric cyberphysical systems in complex manufacturing processes: A comprehensive overview of the global patent profile using collective intelligence. Complexity.

[21] Ballor J.P., McClain O.L., Mellor M.A., Cattaneo A., Harden T.A., Shelton P., Martinez E., Narushof B., Moreu F., Mascareñas D.D.L., Barthorpe R. (2019). Augmented reality for next generation infrastructure inspections. Model Validation and Uncertainty Quantification.

[22] Angrisani L., Arpaia P., Moccaldi N., Esposito A. Wearable augmented reality and brain computer interface to improve human-robot interactions in smart industry: A feasibility study for ssvep signals. Proceedings of the 26 November 2018, Article number 8548517 of the IEEE 4th International Forum on Research and Technologies for Society and Industry, RTSI 2018.

[23] Kot T., Novák P., Babjak J., Mazal J. (2018). Application of augmented reality in mobile robot teleoperation. Modelling and Simulation for Autonomous Systems.

[24] Kot T., Novák P., Babjak J. Using HoloLens to create a virtual operator station for mobile robots. Proceedings of the 19th International Carpathian Control Conference (ICCC).

[25] Lamberti F., Manuri F., Sanna A., Paravati G., Pezzolla P., Montuschi P. (2014). Challenges, opportunities, and future trends of emerging techniques for augmented reality-based maintenance. IEEE Trans. Emerg. Top. Comput..

[26] Ens B., Byagowi A., Han T., Hincapieé-Ramos J.D., Irani P. Combining ring input with hand tracking for precise, natural interaction with spatial analytic interfaces. Proceedings of the 2016 Symposium on Spatial User Interaction (SUI ’16), ACM.

[27] Lee L., Hui P. (2018). Interaction methods for smart glasses: A survey. IEEE Access.

[28] Vorraber W., Gasser J., Webb H., Neubacher D., Url P. Assessing augmented reality in production: Remote-assisted maintenance with HoloLens. Proceedings of the 13th CIRP Conference on Intelligent Computation in Manufacturing Engineering.

[29] Zhou F., Duh H.B.L., Billinghurst M. Trends in augmented reality tracking, interaction and display: A review of ten years of ISMAR. Proceedings of the 7th IEEE/ACM International Symposium on Mixed and Augmented Reality, ISMAR ‘08.

[30] Kim K., Billinghurst M., Bruder G., Duh H.B.L., Welch G.F. (2018). Revisiting trends in augmented reality research: A review of the 2nd decade of ISMAR (2008–2017). IEEE Trans. Vis. Comput. Graph..

[31] Hassan A., Rahini R., Pappas N., Bregoli I. (2016). Consuming “Innovation″ in tourism: Augmented reality as an innovation tool in digital tourism marketing. Global Dynamics in Travel, Tourism, and Hospitality.

[32] Lee K. (2012). Augmented reality in education and training. TechTrends.

[33] DTPoland, Digital Technology Poland, 2017, Hannover Messe Opening with Chancellor Merkel. https://www.dtpoland.com/2017/04/hannover-messe-opening-with-chancellor-merkel/.

[34] Szajna A., Szajna J., Stryjski R., Sąsiadek M., Woźniak W., Burduk A., Chlebus E., Nowakowski T., Tubis A. (2019). The application of augmented reality technology in the production processes. Advances in Intelligent Systems and Computing, Proceedings of the International Conference on Intelligent Systems in Production Engineering and Maintenance ISPEM, Wroclaw, Poland, 28–29 September 2017.

[35] Bryant R.E., Cheng K.T., Kahng A.B., Keutzer K., Maly W., Newton R., Pileggi L., Rabaey J.M., Sangiovanni-Vincentelli A. (2001). Limitations and challenges of computer-aided design technology for CMOS VLSI. Proc. IEEE.

[36] Hamrol A., Gawlik J., Sladek J. (2019). Mechanical engineering in Industry 4.0. Manag. Prod. Eng. Rev..

[37] Schall J.H. (2010). Switch gears for energy distribution and IT. Generalist bei der Energieverteilung und IT. Schaltschranktechnik. TS 8-Systemplattform fuer nahezu alle anwendungsbereiche. Ind. Anz..

[38] Phoenix Contact GmbH & Co. KG Premiere in Hannover: “Smart Engineering and Production 4.0” Technology Network Presents the Complete Vertical Integration of Data 05.03.2015. https://www.phoenixcontact.com/online/portal/pc?1dmy&urile=wcm:path:/pcen/web/corporate/press/press_information/0309b8af-f6c8-4e1c-8726-4b100d617a2e.

[39] EPLAN Software & Service Smart Engineering and Production 4.0—From the top floor to the shop floor. https://youtu.be/16eUSq2r1r4.

[40] Tao F., Zhang H., Liu A., Nee A.Y.C. (2018). Digital twin in industry: State-of-the-Art. IEEE Trans. Ind. Inform..

[41] (2015b). EPLAN Software & Service GmbH & Co. KG. https://www.pressebox.de/inaktiv/eplan-software-service-gmbh-co-kg/Eplan-Experience-die-ersten-365-Tage/boxid/769262.

[42] Rittal Germany, Rittal at the SPS IPC Drives 2015 in Nuremberg. https://youtu.be/T-Pu1dVp4cI.

[43] EPLAN Software & Service GmbH &, Co. KG (2017). EPLAN Smart Wiring: Simplifying Control Cabinet Wiring. https://www.eplan.de/en/solutions/control-cabinet-and-switchgear-engineering/eplan-smart-wiring/.

[44] Schall J.H., Weichsel T. Method and System for Automated Support of a Connection Process in Particular for Components in a Switch Cabinet or on a Mounting System. https://worldwide.espacenet.com/publicationDetails/biblio?II=0&ND=3&adjacent=true&locale=en_EP&FT=D&date=20181024&CC=EP&NR=3392987A1&KC=A1.

[45] Adaszyński M., Ciebiera K., Diks K., Kozlowski T., Szajna A., Szajna J., Zubowicz C., Zyciak M. EP3460719 (2018) The Device for Identifying Wire Markings and the Method for Identifying Wire Markings. https://worldwide.espacenet.com/publicationDetails/biblio?CC=EP&NR=3460719A1&KC=A1&date=&FT=D&locale=en_EP.

[46] Adaszyński M., Szajna J., Ciebiera K., Diks K., Kozłowski T., Szajna A. PL421368 (2019) Device for Identification of Lead Designations and Method for Identification of Lead Designations. https://worldwide.espacenet.com/publicationDetails/biblio?FT=D&date=20181022&DB=&locale=en_EP&CC=PL&NR=421368A1&KC=A1&ND=1.

[47] Schall J.H., Weichsel T. Method and System for the Automated Support of a Connection Process, in Particular of Components Arranged in a Control Cabinet or on a Mounting System. U.S. Patent Application.

[48] Kato H., Billinghurst M., Poupyrev I., Imamoto K., Tachibana K. Virtual object manipulation on a table-top AR environment. Proceedings of the IEEE and ACM International Symposium on Augmented Reality (ISAR 2000).

[49] Mine M., Brooks F., Sequin C. Moving objects in space: Exploiting proprioception in virtual-environment interaction. Proceedings of the SIGGRAPH ’97, International Conference on Computer Graphics and Interactive Techniques.

[50] Boff K.R., Kaufman L., Thomas J.P. (1986). Handbook of Perception and Human Performance.

[51] Schmalstieg D., Fuhrmann A., Hesina G., Szalavári Z., Encarnação L.M., Gervautz M., Purgathofer W. (2002). The studierstube augmented reality project. Presence Teleoperators Virtual Environ..

[52] Kato H., Billinghurst M. Marker tracking and HMD calibration for a video-based augmented reality conferencing system. Proceedings of the 2nd IEEE and ACM International Workshop on Augmented Reality (IWAR’99).

[53] Quoc V.L., Ranzato M.A., Monga R., Devin M., Chen K., Corrado G.S., Dean J., Ng A.Y. Building high-level features using large scale unsupervised learning. Proceedings of the 29th International Conference on Machine Learning.

[54] Hinton G.E., Osindero S., The Y.W. (2006). A fast learning algorithm for deep belief nets. Neural Comput..

[55] Bengio Y., LeCun Y., Bottou L., Chapelle O., DeCoste D., Weston J. (2007). Scaling learning algorithms towards AI. Large-Scale Kernel Machines.

[56] Billinghurst M., Clark A., Lee G. (2015). A survey of augmented reality. Found. Trends Hum. Comput. Interact..

[57] Duin A.H., Armfield D.A., Pedersen I., Getto G., Labriola J., Ruszkiewicz S. (2019). Human-centered content design in Augmented reality. Content Strategy in Technical Communication.

[58] Sluganovic I., Serbec M., Derek A., Martinovic I. HoloPair: Securing shared augmented reality using microsoft HoloLens. Proceedings of the 33rd Annual Computer Security Applications Conference, ACSAC 2017.

[59] Tredoux C.T., Durheim K. (2004). Numbers, Hypotheses and Conclusions: A Course in Statistics for the Social Sciences.

[60] van Aswegen A.S., Engelbrecht A.S. (2009). The relationship between transformational leadership, integrity and an ethical climate in organisations. SA J. Hum. Resour. Manag..

[61] Haller M., Billinghurst M., Thomas B. (2006). Emerging Technologies of Augmented Reality: Interfaces and Design.

[62] Xue H., Sharma P., Wild F. (2019). User satisfaction in augmented reality-based training using microsoft hololens. Computers.

[63] Flavián C., Ibáñez-Sánchez S., Orús C. (2019). The impact of virtual, augmented and mixed reality technologies on the customer experience. J. Bus. Res..

[64] White J. (2019). Microsoft at MWC Barcelona: Introducing Microsoft HoloLens 2. https://blogs.microsoft.com/blog/2019/02/24/microsoft-at-mwc-barcelona-introducing-microsoft-hololens-2/.

[65] Azuma R.T. (2016). The most important challenge facing augmented reality. Presence Teleoperation Virtual Environ..

[66] Viehberger J., Vorraber W., Eisele P.S., Haas F. (2019). Neue augmented-reality-lösung reduziert stillstandszeit im störfall. VDI-Z Integr. Prod..

[67] Szajna A., Kielec R., Szajna J. The new way of maintenance and service of the production lines with the application of augmented reality and artificial intelligence. Proceedings of the 32nd International Business Information Management Association Conference IBIMA.

[68] Evans W.T. Electrical safety, the NFPA and PLC safety. Proceedings of the ASEE Annual Conference & Exposition.

[69] Khan T., Johnston K., Ophoff J. (2019). The impact of an augmented reality application on learning motivation of students. Adv. Hum. -Comput. Interact..

[70] Deng L., Wang G. (2020). Quantitative evaluation of visual aesthetics of human-machine interaction interface layout. Comput. Intell. Neurosci..

[71] Azuma R.T. (2019). The road to ubiquitous consumer augmented reality systems. Human Behavior and Emerging Technologies, Special Issue: Emerging Technologies: Perspectives from Technology Pioneers.

